# An Update of Phytotherapeutic Advances of Marigold (*Calendula officinalis* L.) in Wound Healing

**DOI:** 10.3390/plants14223497

**Published:** 2025-11-16

**Authors:** Georgia Eirini Deligiannidou, Konstantinos Papadimitriou, Efthymios Poulios, Christos Kontogiorgis, Sousana K. Papadopoulou, Constantinos Giaginis

**Affiliations:** 1 Department of Nutritional Sciences and Dietetics, School of Health Sciences, International Hellenic University, 57001 Thessaloniki, Greece; kpapadimitriou@ihu.gr (K.P.); souzpapa@gmail.com (S.K.P.); 2 Laboratory of Hygiene and Environmental Protection, Department of Medicine, Democritus University of Thrace, 68100 Alexandroupolis, Greece; ckontogi@med.duth.gr; 3 Department of Food Science and Nutrition, School of Environment, University of Aegean, 81100 Myrina, Greece; epoulios@aegean.gr

**Keywords:** calendula, marigold, herb, extract, wound healing, animal, human, nanoparticles

## Abstract

Wounds are disruptions of the dermal layer of the skin caused by physical, chemical, thermal, infectious, or immunological insults. Given the skin’s critical role in maintaining homeostasis and protecting against external threats, prompt and effective wound healing is essential to restore functionality and prevent further complications. Numerous natural products (NPs) have long been employed in wound care due to their antioxidant, anti-inflammatory, antimicrobial, and regenerative properties. Building on this historical and scientific foundation, the present literature review consolidates and critically evaluates recent experimental and clinical evidence on the wound healing potential of marigold (*Calendula officinalis* L.). By focusing on studies published between 2020 and 2025, this review captures the evolving understanding of the plant’s therapeutic applications, particularly in skin regeneration and wound management. For each selected publication, formulation type, intervention strategy, dosage, and key outcomes (such as healing rate, cell proliferation, and modulation of inflammatory markers) were summarized. This synthesis aims to provide an update on current evaluations involving *C. officinalis* and how this plant contributes to dermal repair and to identify promising directions for future research and clinical applications.

## 1. Introduction

**Figure 1:**
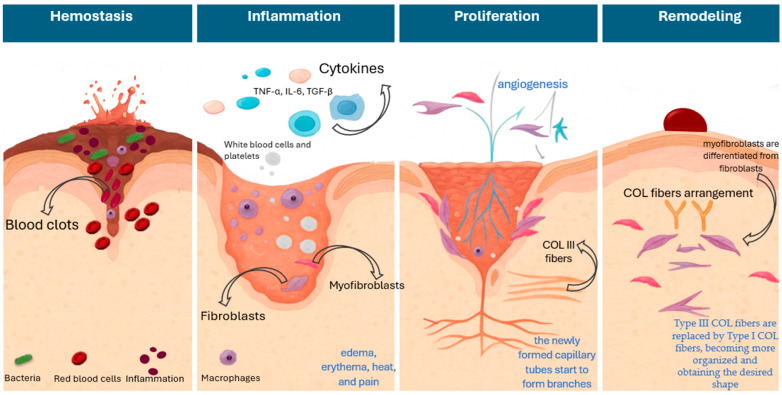
Schematic overview of the stages of wound healing.

A wound is defined as the disruption of the normal anatomical structure and function of a tissue; as such, skin wounds are characterized as injuries of the dermal layer of the skin that can be caused by physical, chemical, thermal, infectious, and/or immunological factors [1]. The skin is the largest organ of the body, which is exposed to various damages, while given its significant role in protecting the human body, the effective and timely restoration of these injuries is essential to regain functionality and prevent more severe complications. In this setting, wound healing (WH), which is characterized by sequential and overlapping stages of hemostasis, inflammation, proliferation, and remodeling, is an important physiological process aiming to restore the structural and functional integrity of the injured area [2,3,4]. Figure 1 presents a schematic overview of the stages of WH.

In brief, during the first stage, the body’s responses include vascular constriction and fibrin clot formation, aiming to stop the bleeding of the wounded area. At this stage, the loss of skin integrity provides fertile ground for microbial colonization [4,5] from organisms such as *Staphylococcus aureus*, coagulase-negative staphylococci, and *Streptococcus pyogenes* (group A streptococcus), which are responsible for the majority of wound infections, as well as Gram-negative species, *Pseudomonas aeruginosa*, *E.coli*, and *Klebsiella pneumoniae* which are also implicated in wound infections [6,7,8]. The inflammatory phase, which follows, is characterized by the infiltration of inflammatory cells into the wound area, along with the presence of edema, erythema, heat, and pain [9]. Several factors are implicated at this stage. During the acute inflammatory response, the arachidonic acid pathway leads to pro-inflammatory eicosanoids, where key enzymes like lipoxygenase (LOX) and cyclooxygenases (COX) are implicated [10,11,12]. In parallel, neutrophils are involved in injury clearance from bacteria and debris, while macrophages continue to digest tissue debris. Macrophages are involved in almost every stage of healing and can recruit inflammatory and fibroblastic cells, thus influencing cells’ proliferation and ultimately tissue remodeling [13,14]. Also, they secrete cytokines and growth factors at the injury site, like epidermal growth factor (EGF), tumor necrosis factor-α (TNF-α), interleukin (IL) -1 and -6, and transforming growth factor-β1 (TGF-β1), which attract immune cells to facilitate tissue repair [15,16,17]. In the proliferative phase, cells start migrating toward the wounded area, and at this stage, the proliferation and migration of fibroblasts, which also produce extracellular matrix (ECM) and collagen (COL) (mainly COL-III [immature form] and COL-I [mature form]), lead to the granulation tissue formation, and the re-epithelialization in the epidermis [18,19]. In brief, the endothelial cells proliferate and migrate to the wound area for the formation of new vasculature, induced by angiogenic factors (e.g., VEGF), which are released by damaged endothelial cells and macrophages, and then the endothelial cells start to develop new micro-vessels from pre-existing ones [20]. Thereby, the newly formed capillary tubes start to form branches necessary for the supply of oxygen and nutrients to the granulation tissue. This is an important step, as the impairment of angiogenesis and blood supply is also related to delayed healing and the presence of chronic wounds [21,22]. Finally, at the remodeling phase, myofibroblasts are differentiated from fibroblasts and start migrating to the wound margins, generating the force required for the contraction of the wound [23,24,25]. Also, Type III COL fibers, which are key constituents of the granulation tissue in the proliferation phase, are gradually replaced by Type I COL fibers, becoming more organized and obtaining the desired shape [23,24,26]. Studies indicate the role of TGF-β1 as a key promoter of COL fibers arrangement through modulation of specific proteolytic enzymes responsible for ECM turnover and homeostasis. Namely, it is known that TGF-β regulates matrix metalloproteinases (MMPs), a group of enzymes responsible for ECM degradation, and thus favors the deposition of ECM proteins and contributes further to ECM reorganization and remodeling [27]. It is worth noting that the increased expression levels of MMP-2 and -7 are identified in the literature as potential predictive biomarkers of delayed wound closure [28].

Since antiquity, man has been tending to cure his wounds, predominantly those inflicted by physical factors, with or without particular access to crude medicinal or pharmacological knowledge. As such, over the centuries, man has turned to natural pharmaceuticals to address this need, and many plants have served in traditional medicine as healing agents in various forms (plant extracts, concoctions, decoctions, topical formulations, or creams) [29]. Natural products (NPs) from medicinal and aromatic plants (MAPs) have been widely used either as extracts or lately as isolated pure compounds, constituting an essential class of therapeutic agents with tremendous potential in treating trauma, due to their chemical constituents (phytochemicals) [30]. A series of NPs have a long history of use in wound care due to their antioxidant, anti-inflammatory, antimicrobial, and essentially wound-healing properties [29]. To date, a large part of the population continues to rely on phytotherapy and traditional medicine to meet their primary health care needs, and a large array of studies are investigating the significant biological role of NPs, based on the indigenous experiences of different cultures. It is also worth noting that, as stated in the Traditional Medicine Strategy 2014–2023 of the World Health Organization (WHO), traditional medicine is a crucial and often underestimated part of health care [31,32].

In the setting of wound healing, a vast variety of plants, such as yarrow (*Achillea millefolium)*, aloe vera (*Aloe vera*), turmeric (*Curcuma longa*), marigold (*Calendula officinalis* L.), tea plant (*Camellia sinensis*), neem (*Azadirachta indica*), and Plantago, have been investigated, particularly in the preclinical setting, and reported to have significant wound healing potential. A key remark is that not as many plants have managed to move on to further evaluations in the clinical setting, and also from those that have managed to do so, only a moderate proportion have demonstrated significant outcomes that can also be comparable to standard treatments. In order to address this particular gap in our understanding of the practical clinical applications of NPs, a recent 2024 systematic review had focused on the clinically investigated herbal formulations in the setting of wound healing. As reported in a previous review, which summarized the outcomes of 26 clinical trials relevant to herbal wound healing formulations, linked to 45 distinct plant species, formulations of St. John’s-wort (*Hypericum perforatum*), Indian pennywort (*Centella asiatica*), *C. officinalis*, and others, documented wound healing attributes such as antimicrobial, anti-inflammatory, and antioxidant [33]. In a similar setting, focusing on the effectiveness of herbal agents compared to a standard medication or placebo in the prevention and therapy of radiodermatitis in breast cancer patients, an overview of randomized clinical trials summarized data from 16 studies involving 1994 patients. Key NPs utilized in the treatment groups were *C. officinalis*, silymarin, and *Aloe vera*. This review and meta-analysis reported that in the setting of radiodermatitis, silymarin showed positive wound healing effects, whereas the efficacy of *C. officinalis* and *Aloe vera* would require more thorough investigations [34]. These results were also similar to previous systematic reviews focusing on radiodermatitis, reflecting on published work that evaluated and compared the effects of herbal formulations, including topical and oral formulations. In these former works, silymarin gel is reported to mitigate the severity of radiodermatitis. Additionally, data on several other plants, like *A. millefolium*, chamomile (*Matricaria chamomilla* L.), and cucumber (*Cucumis sativus*), are also documented, while the efficacy of *C. officinalis* ointment and *Aloe vera* gel has had conflicting overall outcomes [35,36]. A key similarity in these studies is related to the rationale related to the protective effects of these formulations, which was attributed to mechanisms connected with the free radical scavenging, antioxidant, anti-inflammatory, wound healing, and skin protective potential of the plants [36].

*C. officinalis* of the *Asteraceae* family is a popular medicinal plant, widely used in ethnopharmacology for millennia. Currently, the European Medicines Agency (EMA) has maintained the monograph of this plant, stating the medicinal use of *C. officinalis* flowers for minor inflammations of the skin and as an aid in healing of minor wounds (an indication that is strictly related to applications for adults) [37]. The plant contains a variety of bioactive phytochemicals, including flavonoids, triterpenoids, glycosides, saponins, carotenoids, and quinines, which exhibit a range of biological activities, such as anti-inflammatory, anti-cancer, wound healing, and antioxidant properties [38]. Previous investigations of the plant extracts, such as the n-hexanic, ethanolic, and aqueous, were evaluated in human immortalized keratinocytes and human dermal fibroblasts, demonstrating their influence on the inflammatory phase by activating the transcription factor NF-κB and by increasing the amount of the chemokine interleukin-8, with no significant influence in the migration process during the new tissue formation phase (based on the scratch assay) [39]. Additionally, the plant’s hydroethanol extract and its water fraction have been reported to significantly stimulate the proliferation and migration of primary human dermal fibroblasts, while up-regulating the expression of α-smooth muscle actin and connective tissue growth factor, demonstrating faster wound healing in animal models [40]. In the clinical setting, an ointment formulation of the plant has previously been studied for its wound-healing properties against scars following cesarean section. In this 2018 trial involving 72 females categorized into groups of treatment and control, it was reported that using *C. officinalis* ointment considerably increases the speed of cesarean wound healing [41]. In a similar setting, the plant’s extract was evaluated against non-healing venous leg ulcers. It is worth noting that in this study involving a total of 59 patients (19: control, 38: treatment), 72% of the participants in the treatment group achieved complete epithelialisation compared to 32% in the control group, while the average healing time was reduced to 12 weeks with the treatment, and no adverse effects were reported, highlighting the treatment’s safety and efficacy [42]. More recent studies also focused on second-generation lipid nanoparticles, in an effort to document improved outcomes of anti-inflammatory, antioxidant, and antibacterial activities that can aid the plant’s wound healing potential and mitigate toxicity in higher concentrations [43].

Building upon this extensive historical and scientific context, the present literature review aims to consolidate and critically assess recent experimental and clinical findings on the wound healing potential of *C. officinalis*. By focusing on studies published between 2020 and 2025, this review highlights the evolving understanding of the plant’s therapeutic applications, particularly in skin regeneration and wound management, in the setting of both preclinical and clinical evaluations.

## 2. Results

### 2.1. Pre-Clinical Evaluations and Animal Models

**Figure 2:**
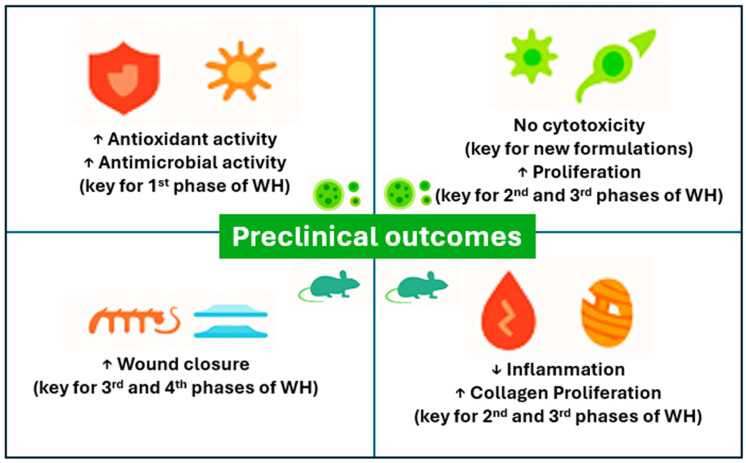
Preclinical outcomes overview.

In the setting of pre-clinical evaluations and animal models, 12 studies have been identified and summarized in this section (Table 1). The studies’ formulations ranged from plant extracts, plant extracts loaded in nanoparticles, hydrogels, or films, and scaffolds, presenting an array of outcomes (Figure 2).

A recent evaluation of *C. officinalis* ethanol extract aimed to document its effectiveness in gingival fibroblast stimulation. The PANsys 3000-a cell culture was utilized, and human fibroblast cells were isolated from the gingival tissue. *C. officinalis* decoction was prepared by boiling 2 g of dried flowers in distilled water at 90 °C for 10 min. After cooling in sterile containers, the extract was filtered, and three concentrations were prepared: a 7% alcohol: 232.5 μL decoction with 17.5 μL ethanol, a 20% alcohol: 200 μL decoction with 50 μL ethanol, and a 100% extract: decoction only. Solid-phase microextraction (SPME) and gas chromatography coupled with tandem mass spectrometry (GC-MS/MS), documented the presence of terpenoids, flavonoids, and other compounds in the extracts, with sesquiterpenes being the major chemical class. Additionally, observation of the gingival fibroblast reported no changes in cell morphology and proliferation. Namely, compared to the positive control group (gingival fibroblasts without extract), no significant differences were observed after 24 and 48 h of culture in cells treated with either 7% or 20% ethanol-containing *C. officinalis* extract, or with the 100% extract without ethanol, demonstrating that cell were able to grow despite the presence of alcohol and potentially due to the plant’s activity, which was able to decrease the alcohol cytotoxic influence on gingival fibroblasts [44].

In the setting of an animal model (male BALB/c mice), a study investigated the wound healing effects of a 5% aqueous *C. officinalis* extract on full-thickness skin injuries. Seventy-two mice inflicted with four symmetrical full-thickness skin wounds were divided into three treatment groups: *C. officinalis*, saline, and untreated controls. Specifically, in this study, 7.5 g of powder from *C. officinalis* flowers were used in the preparation of the extract. The evaluations of the study took place after 14 days of treatment, revealing that treatment was able to significantly accelerate healing by day 7 (wound area (10.29  ±  0.37 mm^2^) compared to saline (12.67  ±  0.70 mm^2^), increasing fibroblast numbers (44 against 33 for *C. officinalis* and saline respectively), and growth factor levels (8.95  ±  0.38, against 6.08  ±  0.92 for *C. officinalis* and saline respectively), while reducing macrophages and inflammatory biomarkers (MMP-2 and MMP-9) in the healing wound. Namely, MMP-2 was reduced from 46.03  ±  1.72 ng/mg (intervention day 1) to 27.68  ±  1.22 ng/mg (intervention day 14), and MMP-9 was reduced from 0.33  ±  0.04 ng/mg (intervention day 1) to 0.18  ±  0.05 ng/mg (intervention day 14), while the reduction in TNF-α was from 14.13  ±  0.58 ng/mg to 7.30  ±  0.63 ng/mg, for day 1 and day 14 respectively, and no statistically significant changes were reported for IL-1 and IL-6 [45].

A recent 2025 study proposes an innovative, nature-based hemostatic biomaterial (skin-adhesive film based on κ-carrageenan, meadow polyfloral honey, and *C. officinalis* flower water-glycerol extract–at concentrations of 5 wt% and 10 wt% of polymer content) for the treatment of wounds in a natural way. The evaluation of this new biomaterial was made on 20 male Wistar rats utilizing the rat tail-cut model. In particular, this new material was able to significantly reduce blood loss (0.1875 ± 0.0732 g) compared to the untreated group (0.7837 ± 0.3319 g), as well as attain faster hemostasis than the control group (355.75 ± 71.42 s against 704.25 ± 85.29 s) [46].

Four studies have evaluated the plant’s potential in the setting of hydrogel formulations. Namely, pullulan/poly (vinyl alcohol) (P/PVA) hydrogels were loaded with the hydroalcoholic extract of *C. officinalis* (5%, 10%, and 20% (*w*/*v*)) by a simple post-loading immersion method. The extract-loaded hydrogels were evaluated for their antioxidant activity, demonstrating up to 70% DPPH radical scavenging after 15 min immersion in buffer solution at pH 5.5. Additionally hydrogels with increasing amounts of *C. officinalis* extract (1.9%, 3.9%, 10.5%) were evaluated for their antimicrobial activity, demonstrating dose-dependent antimicrobial activity as at the highest concentration (10.5%), the inhibition zones reached 15 ± 0.5 mm for *S. aureus*, 14.5 ± 0.2 mm for *E. coli*, and 13 ± 0.1 mm for *P. aeruginosa*, indicating intermediate to susceptible responses [47]. In the setting of cell evaluation (fibroblast cells L929), a hydrogel formulation loaded with nano-liposomes of soy lecithin as a phospholipid containing *C. officinalis* (6 mg dissolved in 50 mL distilled water) has demonstrated no cytotoxicity of the hydrogel in cell proliferation [48]. Similarly, an alginate hydrogel containing *C. officinalis* glycolic extract (10 g of finely powdered plant was used for the formulation of the extract) was evaluated in murine fibroblasts (3T3 cells) and on 50 female Wistar rats. Outcomes regarding the cytotoxicity evaluations revealed increased cell viability in both treatments, with *C. officinalis* being able to enhance the outcomes. Furthermore, in animal testing, the rats were divided into two groups (treated with alginate hydrogel and treated with *C. officinalis*–alginate hydrogel) and inflicted with a wound. After 28 days, the *C. officinalis*–alginate hydrogel (10%) also demonstrated a significant improvement in wound closure, supported by histopathological analysis, showing reduced inflammation, increased macrophage activity, and enhanced collagen deposition [49]. A similar study employed alginate/gelatin hydrogel blended with either simple nanosilver or with nanosilver and plant extracts like aloe vera, turmeric, plantain peel extract, and *C. officinalis* flower petal aqueous extract. The study evaluated the cytotoxicity effects of these new formulations in a fibroblast cell line (V79), demonstrating 79 ± 12% and 50 ± 5% viability for the simple and extract-loaded formulations, respectively (at treatment dose 5 µg/mL in both cases), as well as the antibacterial effects of the hydrogels against *E. coli* and *S. aureus*. The outcomes of this study also demonstrated that the combined effect of nanotechnology and natural extracts was an effective approach for enhancing the wound healing process, as the combination had resulted in 98% scratch closure against 67% in control cells after 72 h [50].

Also, a recent study developed polyacrylamide hydrogels infused with *C. officinalis* extract (10%) for wound healing applications, demonstrating strong biocompatibility and tissue adaptability. Specifically, for the preparation of the extract, 1 kg of the plant was placed in the hydrothanic solution at 70% and left to be macerated for 72 h. The hydrogels evaluated in this study (white hydrogel and hydrogel with *C. officinalis*) exhibited swelling capacities ranging from 715% to 2500% for the white hydrogel and 318% to 1979% for the hydrogel with *C. officinalis*. Additionally, in vivo tests using Wistar rats (divided into 4 groups: saline solution (negative control), SAF-gel^®^ (positive control), 7.2% white hydrogel, and 7.2% *C. officinalis* hydrogel) confirmed enhanced collagen production, skin repair, and no dermal toxicity. Wound size reduction was significant, with the *C. officinalis* hydrogel achieving approximately 50% contraction, which was comparable to SAF-gel^®^, and both hydrogels showed reduced exudate volume [51].

In a similar setting, a chitosan-based hydrogel loaded with silver nanoparticles and *C. officinalis* aqueous extract was evaluated for its antibacterial and wound healing properties. The extract was prepared using 10 g of dried *C. officinalis*, and the nanoparticles were loaded with 2 mL of the extract. The outcomes of the antimicrobial testing of the hydrogels revealed concentration-dependent antibacterial behavior against *E. coli* and *S. aureus*. Additionally, a small evaluation in two diabetic patients resulted in a self-reported positive curative result after use of *C. officinalis*-infused patches every 7 days for two weeks [52]. Also, in the setting of nanoparticles, another approach involved the development of zinc oxide nanoparticles loaded with *C. officinalis* flower aqueous extract (produced using 10 g of dried plant flowers) and their evaluation in antioxidant and wound healing potential. The outcomes of this study documented moderate antioxidant potential as reported by the ABTs and DPPH protocols (scavenging activities of 33.49% and 46.63%, respectively), while the nanoparticles revealed no cytotoxicity at concentrations up to 10 μg/mL (IC_50_ 25.96 μg/mL) in L929 mouse fibroblast cells after 48 h of treatment. Also, the study reported a moderate potential of cell migration and percentage wound closure (69.1%) compared with the untreated cells (64.8%), which were evaluated at 16 h and 12 h after treatment [53].

In the setting of nanofibre-based wound dressing materials that could enhance the process of wound healing, a formulation containing *C. officinalis* of various concentrations 5%, 10% and 15% was evaluated regarding cell viability (L929 mouse fibroblasts), demonstrating non-toxic characteristics and supporting cell attachment and proliferation, and in an in vivo study using male Wistar rats with five full-thickness wounds (divided into groups (untreated, treated with empty nanofiber, treated with extract-loaded nanofiber in the aforementioned concentrations). The outcomes of this study showed that the loaded nanofiber (10%) resulted in a higher wound healing closure rate than the control group on days 7, 14, and 21 after treatment, highlighting the need to further evaluate and standardize the percentage of the extracts used in these investigations [54]. In a similar way, chitosan/polyethylene oxide scaffolds loaded with *C. officinalis* ethanolic extract showed strong antibacterial properties with 96% and 94% reduction in Gram-positive and Gram-negative bacteria, respectively, while also resulting in a significant wound healing potential in the animal testing (rats), demonstrating 87.5% wound closure after two weeks of treatment [55].

### 2.2. Clinical Trials

In the clinical setting, five (5) studies have been identified in evaluating *C. officinalis* (Table 2). Namely, a randomized controlled trial was conducted on 35 patients with bilateral earlobe clefts, and a *C. officinalis* ointment (10%) was used as the selected intervention. The ointment was placed every 12 h on one ear lobe for a period of 7 days, and petroleum jelly was used as the control treatment and placed on the contralateral earlobe. The wound healing potential of the treatment was compared at three different time points, namely at 24 h, 7 days, and 15 days. The outcomes demonstrated a significant difference in the comparison of mean scores of each time point related to the endpoint outcome at 15 days (day 1 (*p* value = 0.525), day 7 (*p* value = 0.324), and day 15 (*p* value < 0.001)) [56].

Also in a clinical setting, 60 burn patients were randomly assigned to receive one capsule (2 g) of *C. officinalis* daily, for two weeks (intervention group) (n = 30), or placebo (control group (n = 30)). The evaluation of the wound status was based on the Bates-Jensen Wound Assessment Tool (BWAT) at three timepoints, namely at 24 h, 7 days, and 15 days. The outcomes demonstrated lower mean total BWAT scores of wound status at the second and third time point for the intervention compared to the control (35.93 and 22.97, against 42.57 and 37.8, respectively), also highlighting a statistically significant decrease in the wound and a subsequent increase in the healing scores comparing days 1 and 15 (*p* > 0.001). Namely, in the intervention group, the range of wound healing changed on days 5 and 15 and was greater (approx. 13 points) than in the control group (approx. 5 points) [57]. In this case placebo was used against the intervention; however, other studies have also used standard treatment to evaluate the added value of a *C. officinalis* extract in the management of wounds. Namely, a clinical trial involving 82 women investigated the efficacy of topical *C. officinalis* lotion (<5% *v*/*v*) against the standard of care (Sorbolene: 10% glycerine in cetomacrogol cream) in reducing the prevalence of radiation-induced dermatitis in women undergoing breast cancer radiotherapy. The treatment duration was set at 6 weeks and included a 6-week follow-up. Interestingly, the outcomes reported no detectable difference in the prevalence of radiation-induced dermatitis grade 2+ between the lotion treatment (53%) and standard care treatment (62%) groups. Also, although the *C. officinalis* lotion resulted in higher scores in the ease of application, general satisfaction, and perceived efficacy scales, mean pain (VAS) scores were lower in the standard treatment (0.85 ± 1.7 against 0.41 ± 0.83, for *C. officinalis* and Sorbolene, respectively) [58]. A *C. officinalis* ointment has also been investigated in the setting of episiotomy in a single-center parallel group randomized trial of women with singleton pregnancies and spontaneous labor at term, in an effort to evaluate its impact on pain levels. The participants (n = 100) were assigned to use either calendula ointment (4 h after the episiotomy and then every 8 h for 10 days) or standard care after episiotomy. In this setting, pain level was self-reported and recorded using the verbal rating scale (VRS), and the outcomes demonstrated that the participants who received the ointment had a significantly lower pain level starting from day two and during all the follow-up [59].

In a similar setting, 3 herbal formulation mixtures (a radiation dermatitis cream mainly based on a combination of *Aloe vera* gel together with *C. officinalis* and *H. perforatum* oil extracts, an containing a combination of beeswax, olive oil, *C. officinalis* and *H. perforatum* oils, and Aloe gel, and a shower gel based on a combination similar t the cream, were evaluated against radiation-induced dermatitis in 59 patients. The participants were asked to use the formulation daily and regularly during radiotherapy and for 2 weeks after treatment ended. The outcomes demonstrated that the majority of patients presented with grade I toxicity in the first weeks of radiotherapy, progressed to grade II, but reverted to grade I toxicity up until the study ended. In addition, no adverse events were recorded during the study, and a total of 94.9% of the patients had Dermatology Life Quality Index scores up to 1, and 66.1% remained in this scale [60].

## 3. Discussion

The present review consolidates recent findings on the wound healing potential of *C. officinalis*, emphasizing its multifaceted therapeutic properties and diverse formulation strategies. Across both preclinical and clinical settings, *C. officinalis* demonstrates promising anti-inflammatory, antioxidant, antimicrobial, and cell-proliferative effects, which are essential for effective wound management. As demonstrated in the literature, the preclinical studies consistently highlight the plant’s ability to modulate key phases of wound healing, including enhancing fibroblast proliferation, collagen deposition, and angiogenesis, while reducing inflammatory markers such as MMP-2, MMP-9, TNF-α, and IL-6. These effects are often attributed to the plant’s rich phytochemical profile, including flavonoids, terpenoids, and carotenoids. It is worth noting that current research is turning into innovative delivery systems to further amplify the plant’s efficacy and reduce toxic events that are common in the use of natural products. Hydrogels, nanofibers, and scaffolds infused with *Calendula* extracts have demonstrated enhanced biocompatibility, antimicrobial activity, and wound closure rates. Inter alia, formulations combining *C. officinalis* with nanotechnology can produce synergistic effects, improving antioxidant capacity and cell migration without cytotoxicity. In the setting of clinical trials, the outcomes reinforce the plant’s therapeutic promise, particularly in managing post-surgical wounds, burns, episiotomies, and radiodermatitis. *C. officinalis* ointments and oral formulations have shown statistically significant improvements in wound healing scores, pain reduction, and patient satisfaction. However, some studies report comparable or even superior outcomes with standard treatments, such as Sorbolene or silver sulfadiazine, underscoring the need for more robust comparative trials.

It must be noted that the variability in formulation type (ointment, capsule, lotion), concentration, and treatment duration across studies complicates direct comparisons and limits generalizability. Moreover, while some trials report clear benefits, others highlight marginal differences or conflicting outcomes, particularly in radiodermatitis management. This inconsistency suggests that *C. officinalis*’s efficacy may be context-dependent, influenced by wound type, patient demographics, and co-administered therapies. Also, despite the encouraging data, several limitations persist, as many preclinical studies rely on small sample sizes and short follow-up periods, while in the setting of clinical trials, lack of blinding, standardized endpoints, or long-term safety assessments are key elements that need to be addressed in future research.

Beyond *C. officinalis*, related species such as three-winged marigold (*Calendula tripterocarpa*) and field marigold (*Calendula arvensis)* have also demonstrated wound healing potential. A recent study evaluating a multi-herbal ointment—including golden mayweed (*Matricaria aurea*), rosemary (*Rosmarinus officinalis*), dyer’s alkanet (*Alkanna tinctoria*), and myrrh—reported superior healing outcomes compared to commercial standards, suggesting synergistic effects among botanicals [61]. Similarly, methanolic extracts of *C. arvensis* accelerated wound contraction and modulated oxidative stress and inflammatory mediators in animal models, providing scientific validation for its traditional use [62]. These findings open avenues for exploring lesser-known *Calendula* species and hybrid formulations, especially in the context of burn injuries and chronic wounds. However, rigorous molecular characterization and standardization remain essential to ensure reproducibility and safety.

Taken together, these studies suggest that *C. officinalis* may serve as a safe, accessible, and effective adjunct in wound care protocols. However, variability in formulation, dosage, and study power highlights the need for further standardized trials and mechanistic exploration.

## 4. Materials and Methods

The aim of this literature review was to identify and synthesize recent clinical and experimental evidence on the wound healing properties of *Calendula officinalis* with emphasis on its application in skin damage. The scope included both in vitro and in vivo studies and randomized controlled trials. A literature search was conducted between March and September 2025 across multiple databases (PubMed, Scopus, and Google Scholar), using combinations of the following keywords: *Calendula officinalis*, *Calendula* extract, wound healing, clinical trial, in vitro, in vivo, burn healing, diabetic wounds, chronic wounds, plant-based therapy, and natural products (Table 3). The initial set publication date was filtered for 2016–2025; however, given the significant number of literature reviews identified between 2016 and 2019, here, we summarize the findings of studies of the last 5 years (2020–2025). From the initial 90 studies identified, only studies that were in English were included in the screening phase of this review.

Included studies met the criteria of focusing on wound healing or skin regeneration using *C. officinalis* and presenting original data from clinical trials, in vivo, or in vitro experiments. The titles and abstracts of identified studies were initially screened for relevance, and full-text articles were retrieved and assessed further before data extraction. From each selected publication, the formulation type, intervention, dose, and key outcomes (e.g., healing rate, fibroblast activity, inflammation markers) were summarized. When available, insights into the safety of applications are also reported. For the selected period and fulfilling the aforementioned criteria, 83 articles were available for screening, 53 were excluded due to study type and time of publication (relevant reviews and studies that did not meet this work’s inclusion criteria -for example exploring mixtures- were retained for potential incorporation to the introduction) or outcomes, and a total of 30 publications were included in the results and discussion sections (4 studies did not meet the criteria to be included in the results since they examined different species of the plant but were retained to be included in the introduction and discussion sections).

## 5. Conclusions

Both preclinical and clinical studies support substantial evidence that *C. officinalis* extracts show promising anti-inflammatory, antioxidant, antimicrobial, and cell-proliferative effects, which are critical for efficient wound management. As a critical future step, the ingredients of *C. officinalis* extracts, which are responsible for their wound healing properties, should be identified. These bioactive ingredients could be used as lead compounds to synthesize relevant synthetic compounds with enhanced wound healing properties. More well-designed clinical studies with adequate participants should also be performed to verify the wound healing properties of *Calendula* extracts and their components.

## Figures and Tables

**Table 1:** Summary of pre-clinical studies.

Study Design	Type of Intervention	Dose (If Available)	Key Outcomes
In vitro (human gingival fibroblasts)	Ethanol extract of *Calendula Officinalis* L. (*C. officinalis)*	7%, 20%, 100% extract	No cytotoxicity; cells proliferated despite alcohol; sesquiterpenes identified as major compounds
Animal model (BALB/c mice)	Aqueous extract of *C. officinalis*	5% extract from 7.5 g powder	Accelerated wound healing by day 7; ↑ fibroblasts & growth factors; ↓ macrophages & MMPs
Animal model (Wistar rats)	κ-carrageenan/honey/*C. officinalis* skin-adhesive film	5 wt% and 10 wt% *Calendula*	↓ blood loss; faster hemostasis vs. control
In vitro (L929 fibroblasts) & antimicrobial assay	Pullulan/PVA hydrogel with hydroalcoholic *C. officinalis* extract	5%, 10%, 20% (*w*/*v*); antimicrobial: 1.9%, 3.9%, 10.5%	Up to 70% antioxidant activity; dose-dependent antimicrobial effect (zones up to 15 mm)
In vitro (L929 fibroblasts)	Hydrogel with nano-liposomes containing *C. officinalis*	6 mg in 50 mL water	No cytotoxicity; supported cell proliferation
In vitro (3T3 fibroblasts) & animal model (Wistar rats)	Alginate hydrogel with *C. officinalis* glycolic extract	10 g plant powder; 10% hydrogel	↑ cell viability; improved wound closure; ↓ inflammation; ↑ collagen & macrophage activity
In vitro (V79 fibroblasts)	Alginate/gelatin hydrogel with nanosilver + *C. officinalis* extracts	5 µg/mL	79% viability (simple); 50% (extract-loaded); 98% scratch closure vs. 67% control
Animal model (Wistar rats)	Polyacrylamide hydrogel with *C. officinalis* extract	10% extract from 1 kg of plant	50% wound contraction; ↑ collagen; no dermal toxicity; ↓ exudate volume
In vitro & small-scale human evaluation	Chitosan hydrogel with silver nanoparticles + *C. officinalis*	2 mL extract from 10 g dried plant	Antibacterial vs. *E. coli* & *S. aureus*; 2 diabetic patients reported positive healing
In vitro (L929 fibroblasts) & animal model (Wistar rats)	Zinc oxide nanoparticles with *C. officinalis* extract	10 g dried flowers; up to 10 µg/mL	Moderate antioxidant activity; no cytotoxicity; 69.1% wound closure vs. 64.8% control
In vitro (L929 fibroblasts) & animal model (Wistar rats)	Nanofiber wound dressing with *C. officinalis*	5%, 10%, 15%	Non-toxic; 10% extract showed best healing on days 7, 14, 21
Animal model (rats)	Chitosan/polyethylene oxide scaffold with *C. officinalis*	Not specified	96% (Gram+) & 94% (Gram−) bacterial reduction; 87.5% wound closure in 2 weeks

**Table 2:** Summary of clinical interventions.

Study Design	Population	Intervention	Control	Dose & Duration	Key Outcomes
RCT (bilateral earlobe clefts)	35 patients	*C. officinalis* ointment (10%)	Petroleum jelly	Applied every 12 h for 7 days	Significant improvement at day 15 (*p* < 0.001); no difference at day 1 or 7
RCT (burn patients)	60 patients	*C. officinalis* capsule (2 g/day)	Placebo	Daily for 2 weeks	Lower BWAT scores at day 7 & 15; greater healing range (13 vs. 5 points); *p* > 0.001
RCT (breast cancer radiotherapy)	82 women	*C. officinalis* lotion (<5% *v*/*v*)	Sorbolene cream	Applied during 6-week treatment + 6-week follow-up	No difference in dermatitis grade 2+; *Calendula* rated higher in satisfaction; Sorbolene had lower pain scores
RCT (episiotomy recovery)	100 women	*C. officinalis* ointment	Standard care	Applied 4 h post-episiotomy, then every 8 h for 10 days	Lower self-reported pain from day 2 onward across all follow-up points

**Table 3:** Methodology steps in the identification and evaluation of articles.

Step	Description
Review Objective	Identify recent evidence on *Calendula officinalis* in wound healing
Databases	PubMed, Scopus, Google Scholar
Search Period	March–September 2025
Keywords	Calendula officinalis, wound healing, clinical trial, in vitro, in vivo, etc.
Date Range	2016–2025 (final selection: 2020–2025)
Language	English only
Inclusion Criteria	Original data from clinical trials, in vivo, or in vitro studies on wound healing
Screening Process	Title/abstract screening → full-text review → data extraction
Data Extracted	Formulation type, intervention, dose, healing outcomes, safety

## Data Availability

The data of the present study are available upon request to the corresponding author.
